# Does preparation generate the cost of task switching? A recipe for a switch cost after cue-only trials

**DOI:** 10.1007/s00426-025-02107-2

**Published:** 2025-03-24

**Authors:** Motonori Yamaguchi, Rachel Swainson

**Affiliations:** 1 Department of Psychology, University of Essex, Colchester, UK; 2 School of Psychology, University of Aberdeen, Aberdeen, UK

## Abstract

A switch cost can be observed in cued task-switching on trials that follow a *cue-only trial*, which presents a task cue indicating a task to be performed but does not present a target stimulus to be responded to. This finding has provided important implications as to the source of the performance cost that emerges when switching tasks. However, cue-only trials differ from completed trials (for which the target occurs and is responded to) in several task parameters, and there are a few untested assumptions about a task-switch cost after cue-only trials, which restricted the conditions under which cue-only trials have been used. The present study first examined whether a switch cost emerged after cue-only trials when cue-only trials were matched with completed trials in as many task parameters as possible, and found that an expected switch cost following cue-only trials was absent in response time. In the subsequent six experiments, we explored critical task parameters to obtain a switch cost after cue-only trials. The present results indicate that the use of a short preparation interval was an important factor and that the switch cost was more short-lived and dissipated more quickly after cue-only trials than after completed trials. These outcomes are consistent with the proposal that there are at least two sources of a task-switch cost, one that originates from processing a task cue and another that originates from performing a cued task. Early processes of task preparation (e.g., cue or task identification) may be sufficient to produce the switch cost after cue-only trials, but response-related processes might generate a more persistent switch cost.

## Introduction

Preparation is the key to success in life. As Thomas Edison famously said (*Edison Quotes*, n.d.), good fortune often happens when opportunity meets with preparation. There would be no shortage of episodes that highlight the importance of preparation in our lives. This is also true about everyday chores, such as fixing dinner or receiving a delivery: people usually perform a task more efficiently and are more likely to be successful when they are prepared for it than when they are not. An experimental paradigm that arguably demonstrates the impact of preparation on task performance is the *cued task-switching* procedure (Meiran, 1996). In this procedure, participants perform two (or sometimes three) tasks that are intermixed randomly in a series of trials. A task cue is presented at the beginning of each trial to indicate which task should be performed on that trial. Participants would then prepare for the cued task and respond to the target stimulus according to the prepared task rules (e.g., Monsell, 2003). A typical finding in this procedure is that a response to the target is faster when the current task is the same as the one that is just performed on the preceding trial (*task-repeat trial*) than when it is different from the previous task (*task-switch trial*). The performance difference between task-repeat and task-switch trials is called the *switch cost* (see Kiesel et al., 2010; Vandierendonck et al., 2010, for reviews).

It has been shown that a switch cost can also be obtained when the preceding trial only presents a task cue that indicates a task to perform without the actual target to which a response is to be made (*cue-only trial*; Lenartowicz et al., 2011). This switch cost after cue-only trials is suggested to reflect the effect of mere preparation for the cued task because the cued task is not overtly performed. However, there have been only few other studies that have used cue-only trials (Brass & von Cramon, 2004; Desmet et al., 2012; Prosser et al., 2023; Swainson et al., 2017, 2021, 2024), and several untested methodological choices were made based on the assumption that they were necessary to obtain a switch cost after cue-only trials. These choices have restricted the conditions under which a switch cost after cue-only trials has been examined, and, thus, the nature of the switch cost after cue-only trials is yet to be understood. Therefore, the present study examined various untested assumptions to determine the conditions under which a switch cost is (or is not) obtained after cue-only trials.

### What is a cue-only trial, and why does it matter?

To our knowledge, the first study that used cue-only trials in cued-task switching is that by Brass and von Cramon (2004). They presented two task cues in a sequence (double-cue trials) for which only the second one was followed by the target. They found a cost of switching the task cues both when the two cues indicated the same task and when they indicated different tasks. More recently, Lenartowicz et al. (2011) demonstrated a switch cost after cue-only trials in response to an earlier finding that a switch cost was absent when participants withheld responding to the target on a preceding trial (i.e., a nogo trial; Schuch & Koch, 2003). In the go/nogo method used by Schuch and Koch, participants were first presented with a task cue, followed by a target along with an additional go or nogo signal (e.g., high- or low-pitch tones); participants would then decide whether to respond to the target according to the signal (also see Hoffman et al., 2003). The researchers examined the switch cost on go trials that followed either another go trial or a nogo trial, and they found that a switch cost was obtained after go trials but not after nogo trials. Following this finding, the researchers further showed that: (1) the switch cost was also absent when the nogo signal was replaced with a neutral target that was not assigned to any response (so no response could be activated); (2) the cost of repeating the task performed two trials ago (known as the N-2 repetition cost, which is attributed to inhibition of a previous task-set when switching tasks; see Koch et al., 2010, for a review) was also absent after nogo trials; (3) the N-2 repetition cost was still absent when participants pressed both of the two response keys in response to the nogo signal (double-press trials) rather than selecting one of the keys (also see Koch & Philipp, 2005, who reported a reduced switch cost after double-press trials); and (4) the N–2 repetition cost was still absent when the lengths of go and double-press trials were matched. From these results, it was concluded that the lack of response selection on a nogo trial was responsible for the absence of task-switch and N–2 repetition costs after nogo trials. They suggested that multiple task-sets can be activated simultaneously, but the task-sets that are irrelevant to the current task become inhibited in the process of selecting a response to the target (Schuch & Koch, 2003) or executing the selected response (Philipp et al., 2007). These mechanisms yield an N-2 repetition cost and a switch cost, but neither response selection nor execution occur on nogo trials, eliminating the cost of task switching after nogo trials. An important message of these explanations is that task preparation is not sufficient to produce an N–2 repetition cost or a switch cost on the following trial. For example, Schuch and Koch (2003) concluded that “interference results only from changing demands on action selection; there is no interference resulting from preparing for different tasks in close succession” (p. 104).

**Fig. 1:**
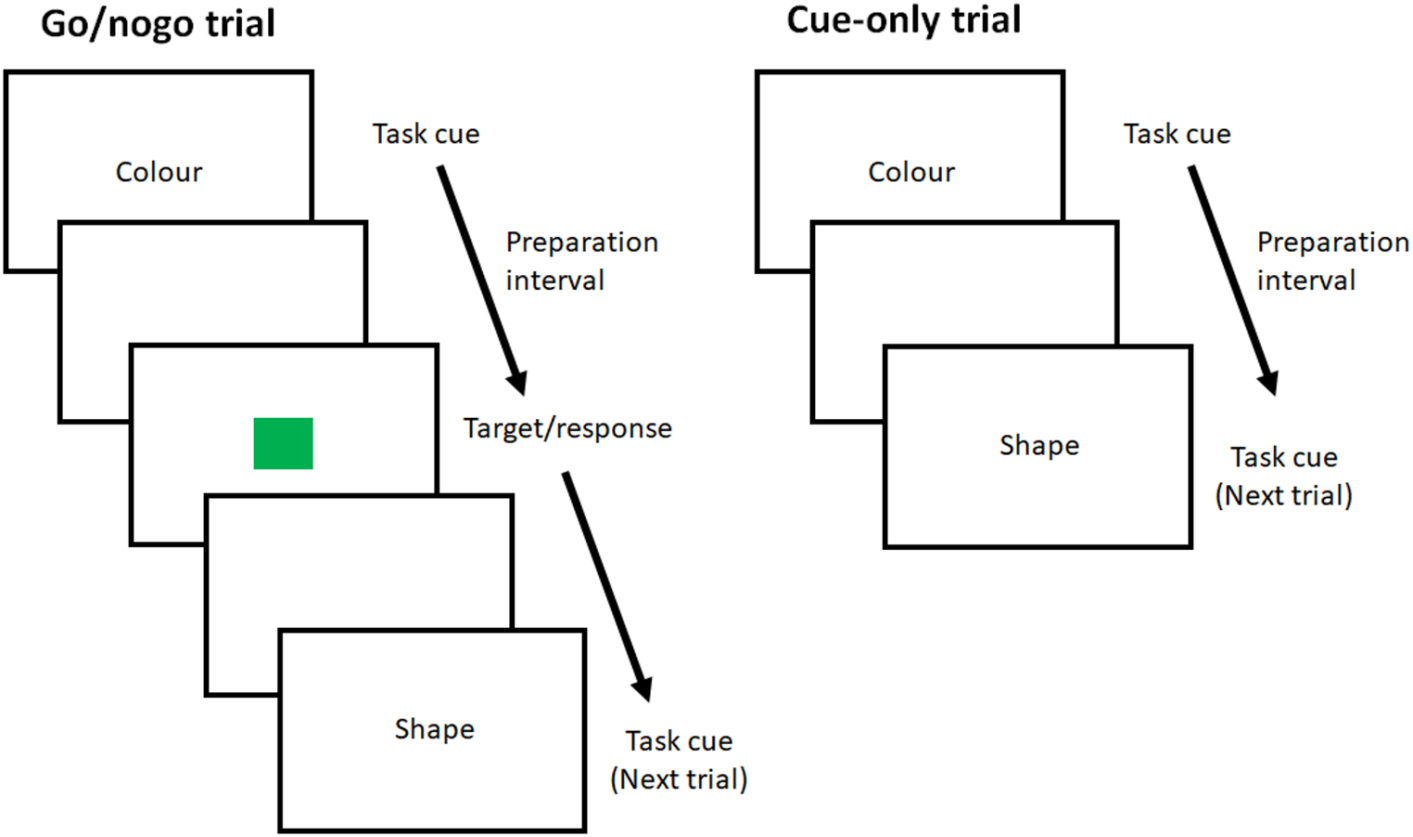
Illustrations of go/nogo and cue-only trials. For go/nogo trials, an additional stimulus (e.g., an auditory tone) may be presented with the target as a go or nogo signal, which is not shown in the illustration

Lenartowicz et al. (2011) adopted the go/nogo procedure of Schuch and Koch, but in Experiment 2, they replaced nogo trials with cue-only trials (see Fig. 1 for illustrations of the two trial types; note that the tasks, cues and target stimuli shown are not identical with those used by Lenartowicz et al.). Unlike nogo trials, cue-only trials were terminated without presenting the target. Because there was no target, response selection or execution could not occur on cue-only trials. However, Lenartowicz et al. found a significant switch cost after cue-only trials and suggested that task preparation was sufficient to obtain a switch cost on the next trial. They considered two possible reasons why the switch cost was absent after nogo trials. The first possibility was, as Kleinsorge and Gajewski (2004) also suggested, that because a proportion of trials were nogo trials, participants might have been discouraged from advance preparation of the cued task at onset of the task cue. However, Lenartowicz et al. pointed out that this explanation did not account for Schuch and Koch’s finding that the switch cost decreased with increased intervals between the task cue and the target, which reflected the preparation of the incoming task during the intervals. The second possibility was that the cued task was prepared on nogo trials, but nogo signals interfered with the prepared task-set, eliminating the advantage of repeating the same task. Lenartowicz et al. favored the second explanation and proposed that removing nogo signals (as in their cue-only trials) would reveal effects of task preparation. We have also found recently that selectively withholding response to the target required response selection but still eliminated a task-switch cost on a subsequent trial (Yamaguchi & Swainson, 2024), supporting the idea that nogo signals interfere with a previously activated task-set. Therefore, finding a switch cost after cue-only trials has provided an important indication that task preparation generates a subsequent switch cost.

The task-switch cost is thought to reflect the time to reconfigure a task-set for the incoming task (Meiran, 1996) or the effect of interference with the activation of a task-set from another task-set used on the preceding trial (Allport & Wylie, 2000). Such reconfiguration or activation of a task-set could be considered to occur during preparation for the incoming task. However, other accounts of switch cost do not assume such preparatory processes upon the presentation of a task cue. For example, Logan and Bundesen (2003) proposed a compound-cue retrieval model in which the task cue and the target form a compound cue to retrieve the correct response required on a given trial (but see Forrest et al., 2014). This model does not require a task-set to be activated or reconfigured to perform the cued task prior to processing the target. A more recent model also emphasizes episodic retrieval of previously encountered trial features that either facilitate or interfere with performance on the current trial (Schmidt et al., 2020). This model is similar to the idea of the compound-cue retrieval model of Logan and Bundesen, but it also extends the idea of compound cues to include features other than the task cue and target stimuli (e.g., previous response or decision). Considering these competing views of task switching, what is involved in the preparatory process at the presentation of a task cue is still a subject of debate, and it raises the question of what underlies a switch cost after cue-only trials.

Recent studies using a backward inhibition design showed that an N–2 repetition cost still emerged when a cue-only trial occurred on the N–2 trial (two trials before the current trial; Berger, Koch et al., 2024) or on the N–1 trial (i.e., one trial before the current trial; Prosser et al., 2023), in the latter case affecting only cue responses (reflecting task identification) rather than target responses. The former finding suggests that the activated task-set for a cue-only trial was inhibited by the following completed trial, whereas the latter suggests that a cue-only trial inhibited the task used on the preceding trial (but see Grange et al., 2017, for an alternative interpretation of the N–2 repetition cost). Swainson et al. (2017) used cue-only trials as in Lenartowicz et al.’s (2011) study. In their Experiment 3, they randomized stimulus-response mappings for each task across trials and informed participants of these mappings only after seeing the target on each trial. Thus, a task cue would signal a specific task (i.e., either the color task or the shape task), but participants could not define the specific set of stimulus-response mappings required for the task on each trial until 200 ms after the target appeared. If the switch cost after cue-only trials was due to a retrieval of stimulus-response mappings, this procedure would not yield a switch cost after cue-only trials because these mappings were not revealed on cue-only trials. On the other hand, if the identification of a cued task was a sufficient preparation to yield a cost of switching tasks, a switch cost would still be obtained after cue-only trials. Swainson et al.’s results were consistent with the latter: a switch cost was obtained after cue-only trials even when participants could not retrieve a specific set of stimulus-response mappings at the onset of a task cue. Thus, task identification might be sufficient to obtain a switch cost after cue-only trials. Because task identification would require task-cue encoding, a switch cost after cue-only trials could potentially only involve a cue-switch cost rather than a task-switch cost (Logan & Bundesen, 2003), but previous studies have also shown that a switch cost is obtained after cue-only trials when a cue-repetition is excluded (e.g., Swainson et al., 2017, 2021, 2024). Although the finding of a switch cost after cue-only trials is suggestive of the source of a switch cost after cue-only trials, more scrutiny is warranted to understand the nature of the switch cost that can be driven in the absence of performance. To this end, it is important to probe factors that influence a switch cost after cue-only trials and to compare it to a switch cost after trials in which the cued task is performed (which we call *completed trials* because a task is completed on these trials).

### The present study

In response to the proposal that the switch cost depended on response selection for a target but not on preparation for the cued task (Schuch & Koch, 2003), Lenartowicz et al., (2011) demonstrated that a switch cost could be obtained without a target having been shown on the preceding cue-only trial. They concluded that preparation was enough to generate a switch cost (also see Brass & von Cramon, 2004). If preparation on cue-only trials generates a subsequent switch cost, it makes sense to ask whether preparation (rather than response selection) is responsible for producing the switch cost following completed trials too. If so, then the switch cost following cue-only trials should be similar to that following completed trials (Swainson et al., 2024). Nevertheless, there were several untested assumptions as to what might be necessary to obtain a switch cost after cue-only trials that led to restricted conditions under which cue-only trials were used in the previous studies, and these restrictions introduced systematic differences between cue-only and completed trials. As a switch cost after cue-only trials would depend on advance preparation of a cued task before a target occurs, researchers have used conditions that encourage advance preparation. For example, it was thought that a long preparation interval was important for task preparation to occur on cue-only trials. Hence, cue-only trials with long preparation intervals were prioritized in the analyses (Lenartowicz et al., 2011) or cue-only trials only ever involved a long preparation interval (Swainson et al., 2017, 2021). It has also been usual practice to make cue-only trials less frequent than completed trials because it was thought that frequent cue-only trials discourage advance preparation (Lenartowicz et al., 2011; Swainson et al., 2017, 2021). There is also a systematic structural difference between cue-only and completed trials: because cue-only trials omit the target, these trials terminate much sooner than completed trials. Therefore, the primary motivation of the present study was to examine whether a switch cost could be obtained after cue-only trials when cue-only trials were matched closely with completed trials in as many aspects as possible.

**Fig. 2:**
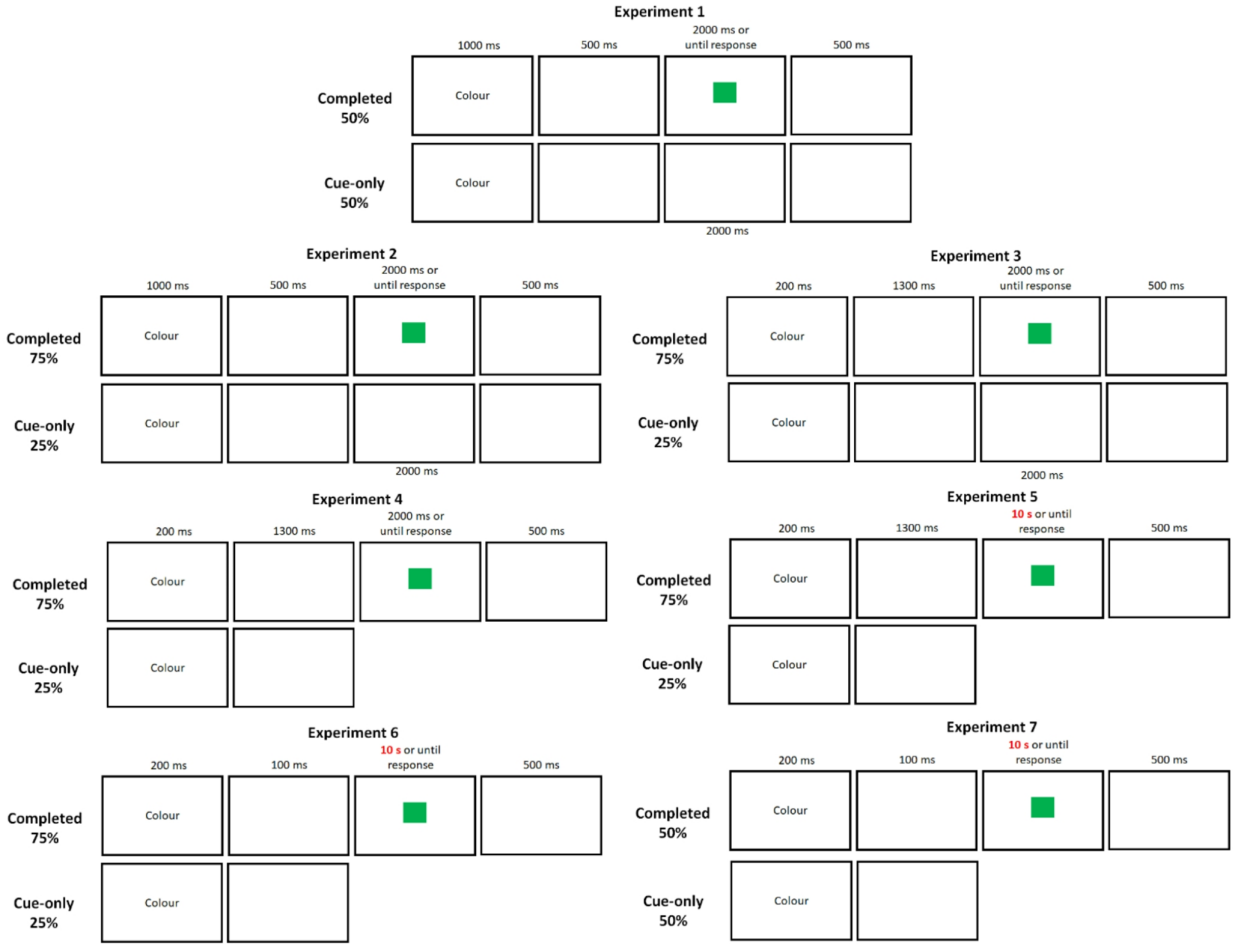
The sequences of events on completed and cue-only trials in experiments 1–7

In Experiment 1, we first equated cue-only and completed trials in terms of their frequency, their ability to occur consecutively, and their total length, such that these two trial types only differed in terms of the presence of a target stimulus and, therefore, performance of the cued task. To provide a preview of the results, we obtained a switch cost after completed trials but failed to obtain a significant switch cost in response time (RT) after cue-only trials. Given the robustness of switch cost in RT after completed trials, this outcome would be a rather surprising result under the assumption that the switch cost after cue-only trials originated from the same source as that which generated the switch cost after completed trials. Therefore, the subsequent experiments (Experiments 2–7) explored various task parameters and examined the underlying processes that are responsible for a switch cost after cue-only trials. Figure 2 illustrates the sequence of events on a trial in the seven experiments.

An overview of the present study is as follows: It has previously been suggested that both the proportion of nogo trials (Kleinsorge & Gajewski, 2004) and the duration of a task cue (Verbruggen et al., 2007) can influence the advance preparation of a cued task, such that a high proportion of nogo trials or a long cue duration discourage advance preparation. If either the equal proportion of cue-only and completed trials or the long presentation of a task cue had been responsible for the absence of switch costs in RT following cue-only trials in Experiment 1, such a cost should be obtained after cue-only trials if cue-only trials are made less frequent (Experiment 2) or when the presentation of a task-cue is made shorter (Experiment 3). It has also been suggested that the cue-cue interval could influence the size of the switch cost after cue-only trials (Swainson et al., 2021). Thus, Experiment 4 shortened the cue-cue interval following cue-only trials by removing the blank display after the preparation interval of cue-only trials that occurred in place of the target on completed trials in Experiment 1. Furthermore, previous studies that focused specifically on switch costs after cue-only trials (e.g., Lenartowicz et al., 2011; Swainson et al., 2017, 2021; but see Brass & von Cramon, 2004) did not use a response deadline, whereas Experiment 1 of the present study did. Experiment 5 used a longer response deadline (10 s), which essentially removed the time pressure to make a response quickly. Finally, in the last two experiments (Experiments 6 and 7), the preparation interval was reduced to 300 ms on all trials: In Experiment 6, the proportion of cue-only trials was smaller than that of completed trials, and in Experiment 7, there was an equal proportion for the two trial types. Overall, the results of these experiments provided new insights into task parameters that were, or were not, critical for a switch cost to occur after cue-only trials. Incidentally, they also suggested that there are differences between the factors that affect switch costs after cue-only trials and those that affect switch costs after completed trials, which implied that switch costs may originate from different cognitive processes after cue-only and completed trials.

## General method

### Participants

All experiments were conducted online when the restrictions due to the COVID-19 pandemic were still in place. Participants were recruited from the Prolific platform (https://www.prolific.co/). With a fully within-subject design, 34 participants were required to achieve a statistical power of 0.8 to detect a medium effect size (*f* > 0.25 in G*Power; Faul et al., 2007). We aimed to recruit more than 34 participants, with the following inclusion criteria: they were residents of the UK or the United States, who reported being fluent in English, having normal or corrected-normal vision and normal color vision, having no ongoing cognitive impairment and taking no antipsychotic medication, and being between 18 and 40 years old. They also had to have completed more than 20 studies with an approval rate of higher than 95% on Prolific, and they were not allowed to participate if they had completed any of the other experiments reported here. Participants were also excluded when their overall error rates in the task were greater than 30%. In Experiment 1, 40 participants were recruited, but one participant was excluded for a high error rate, leaving a total sample size of 39 (18 females, 20 males, 1 non-binary; mean age = 31.26, SD = 6.19, range = 21–40). In Experiment 2, 39 participants (23 females, 16 males; mean age = 29.51, SD = 7.74, range = 19–40) were recruited. In Experiment 3, 41 participants were recruited, but one participant was excluded for a high error rate, leaving 40 participants (25 females, 15 males; mean age = 30.90, SD = 6.44, range = 19–40). In Experiment 4, 39 participants were recruited (22 females, 17 males; mean age = 32.36, SD = 5.91, range = 19–40). In Experiment 5, 40 participated were recruited, but one participant was excluded for a high overall error rate, leaving 39 participants (20 females, 19 males; mean age = 31.28, SD = 5.96, range = 20–39). In Experiment 6, 41 participants were recruited, but one participant was excluded for a high overall error rate, leaving 40 participants (20 females, 16 males, 4 non-binary; mean age = 29.05, SD = 6.46, range = 18–40). In Experiment 7, 41 participants were recruited, but two participants were excluded for a high error rate, leaving 39 participants (18 females, 20 males, 1 non-binary; mean age = 31.85, SD = 5.23, range = 20–40). All participants provided informed consent before data collection commenced. The experimental protocol was reviewed and approved by the Research Ethics Committee of the University of Essex.

### Transparency and openness

The data, analysis scripts, and experimental programs are available on the OSF project page (https://osf.io/9p2ws/). We reported how we determined our sample size in the Participants section above. The design and its analyses were not pre-registered. All data were collected between May 2022 and August 2022.

### Apparatus and stimuli

All experiments were controlled by the Inquisit software (ver. 6; Millisecond Software, LLC.) that ran on participants’ own computers. Tablet computers and smart phones were not allowed, and the experimental program worked only with Mac or Windows PCs. Participants were required to install a player application to run the experiment program, which took over the control of participants’ computer during the experiment. The sizes of stimuli were adjusted, and thus varied, according to participants’ monitor size. The following measurements were taken from a screen resolution of 1920 × 1080 in a 13-in laptop monitor. The target stimuli were green and red squares (2 cm each side) and diamonds (the same squares tilted 45°). They appeared at the center of the screen. The task cues were the word “COLOUR” for the color task and the word “SHAPE” for the shape task, which were presented in the Arial font and appeared 3.5 cm above the screen center. These task cues were also used in our previous studies (e.g., Yamaguchi & Proctor, 2011; Yamaguchi & Swainson, 2024; Yamaguchi et al., 2017). Responses were made by pressing the ‘S’ and ‘L’ keys on the keyboard.

### Procedure

Participants first read the task instructions and were informed that there were two types of trials, one that presented both the task cue and the target (completed trial) and the other that presented the task cue without the target (cue-only trials). They were told to respond to the target as quickly and as accurately as they could on completed trials but do nothing and wait for the next trial on cue-only trials. Participants then downloaded the Inquisit Player to their computers and started the experiment. They were instructed on the assignment of target colors and shapes to the two response keys before practice trials started. This assignment was randomly determined for each participant and remained fixed throughout a session. The two tasks required classifying the color (red vs. green) or shape (square vs. diamond) of the target and pressing one of the two response keys. These two tasks occurred randomly with an equal probability as well.

There was one block of 12 practice trials for which all trials were completed trials, followed by another block of 24 practice trials in which completed and cue-only trials were randomly intermixed. Participants then performed four blocks of 96 test trials that also included completed and cue-only trials in a random order. The sequences of events on cue-only and completed trials in Experiments 1–7 are illustrated in Fig. 2. The task parameters for the seven experiments are also summarized in Table 1.

**Table 1:** Summary of task parameters and presence/absence of switch costs in experiments 1–7

	Experiment						
	1	2	3	4	5	6	7
Proportion of trials completed/cue-only trials	50% / 50%	75% / 25%	75% / 25%	75% / 25%	75% / 25%	75% / 25%	50% / 50%
Task cue duration	1000 ms	1000 ms	200 ms	200 ms	200 ms	200 ms	200 ms
Blank after task cue	500 ms	500 ms	1300 ms	1300 ms	1300 ms	100 ms	100 ms
Response deadline on completed trials	2000 ms	2000 ms	2000 ms	2000 ms	10,000 ms	10,000 ms	10,000 ms
Cue-only trials terminated after blank following a task cue	No	No	No	Yes	Yes	Yes	Yes
Switch cost after completed trials: RT	Present	Present	Present	Present	Present	Present	Present
Switch cost after completed trials: PE	Present	Present	Present	Present	Present	Present	Present
Switch cost after cue-only trials: RT	Absent	Absent	Absent	Absent	Absent	Present	Present
Switch cost after cue-only trials: PE	Present	Absent	Absent	Absent	Absent	Absent	Absent

#### Experiment 1

In this experiment, completed and cue-only trials occurred with an equal probability. For completed trials, each trial started with a task cue that stayed on the display for 1000 ms, which was followed by a blank display lasting for 500 ms. A target then appeared in the screen center until a response key was pressed or, when no response was made, for 2000 ms. For correct responses, there was a 500-ms blank display before the next trial started. For an incorrect or no response, the error message “Error!” appeared in the screen center for 2000 ms (this long duration of error feedback was intended to discourage participants from responding randomly). For cue-only trials, the timing of events was essentially the same as those for completed trials, except that the target did not show up and instead a blank display after the task cue was prolonged for 2000 ms (to match with the response deadline on completed trials). RT was measured as the interval between onset of the target stimulus and a depression of a response key.

#### Experiment 2

This experiment only differed from Experiment 1 in the number of completed and cue-only trials, which occurred with 0.75 and 0.25 probabilities, respectively.

#### Experiment 3

The experiment included the following changes from Experiment 2: The task cue was presented for 200 ms, instead of 1000 ms, and the following blank display appeared for 1300 ms, instead of 500 ms. Thus, the sum of the durations of a task cue and the following blank display was 1500 ms, identical with that of Experiments 1 and 2. Note that cue-only trials still included an additional blank display following this preparation interval to match the maximum duration of completed trials.

#### Experiment 4

The only change from Experiment 3 was that cue-only trials ended after the 1300-ms blank display following the task cue, without the additional blank display.

#### Experiment 5

The response deadline was increased to 10 s, which was considered to be more than enough time within which to make a slow response. The experiment was identical with Experiment 4 in other respects.

#### Experiment 6

The duration of the blank display following a task cue was reduced to 100 ms, making the preparation interval 300 ms (the 200-ms task-cue duration plus the 100-ms blank).

#### Experiment 7

The only difference from Experiment 6 was that cue-only and completed trials occurred with the same probability, as in Experiment 1.

## Results

The data were analyzed in terms of mean RT for correct responses and percentage of error trials (PE) on completed trials (i.e., 50% of all trials for Experiments 1 and 7 and 75% for Experiments 2–6) as there were no responses on cue-only trials. Error trials included those on which participants pressed an incorrect response key, and did not include trials with no response, which were discarded before the data analysis. Trials were also discarded before the analysis if RT was less than 200 ms or if the preceding trial was an error trial^1^. The first trial in each block was not counted as there was no previous trial. We report the proportions of discarded trials in each experiment below.

**Fig. 3:**
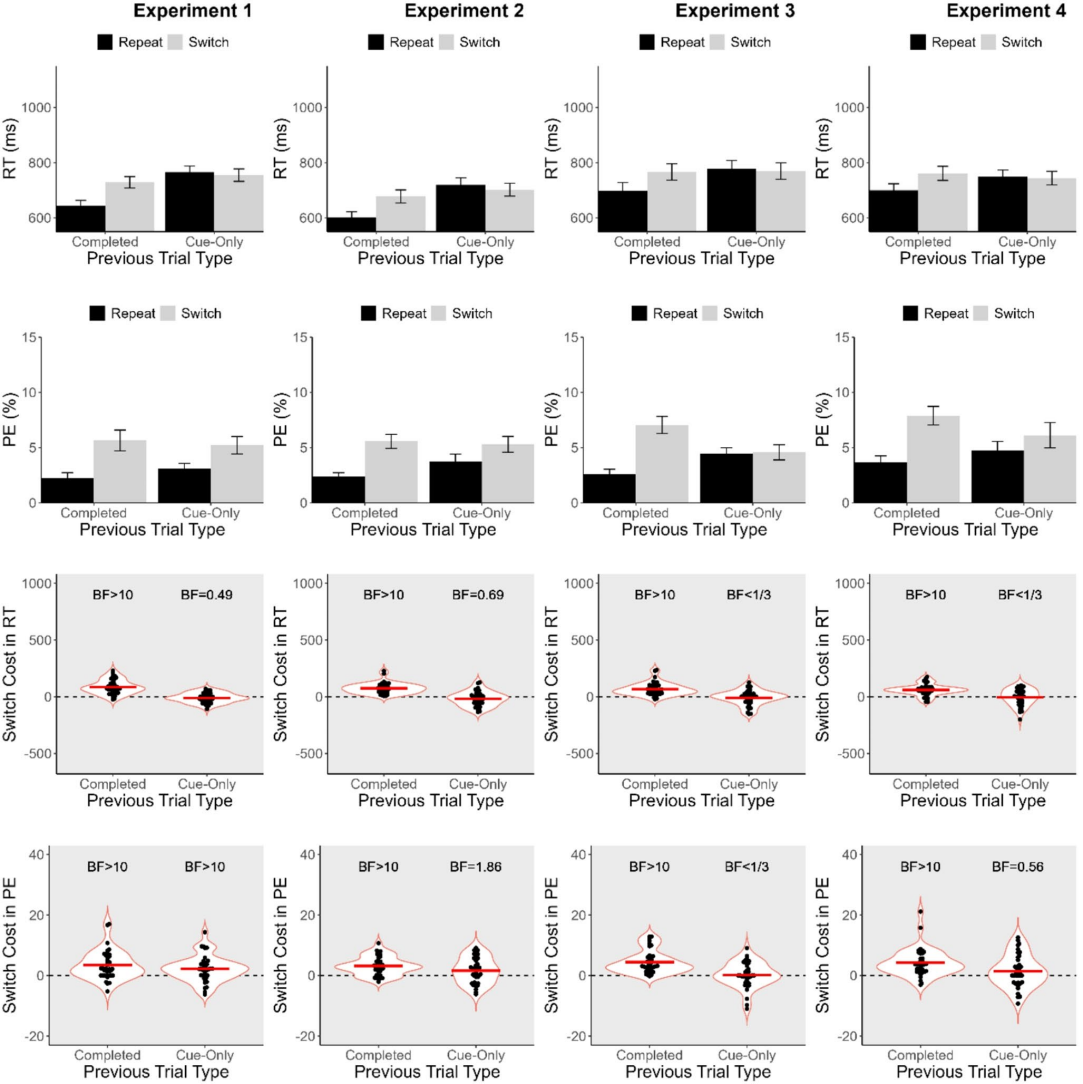
Mean response time (RT), percentage error (PE), switch cost in RT, and switch cost in PE in Experiments 1–4. Error bars in the bar graphs represent one standard error of the means. Horizontal lines within the violin plots represent the grand means, and BFs are the corresponding Bayes factors. BF > 10 or > 3 indicates the presence of a switch cost; BF < 1/10 or < 1/3 indicates evidence for the absence of a switch cost; everything else indicates inconclusive evidence

**Fig. 4:**
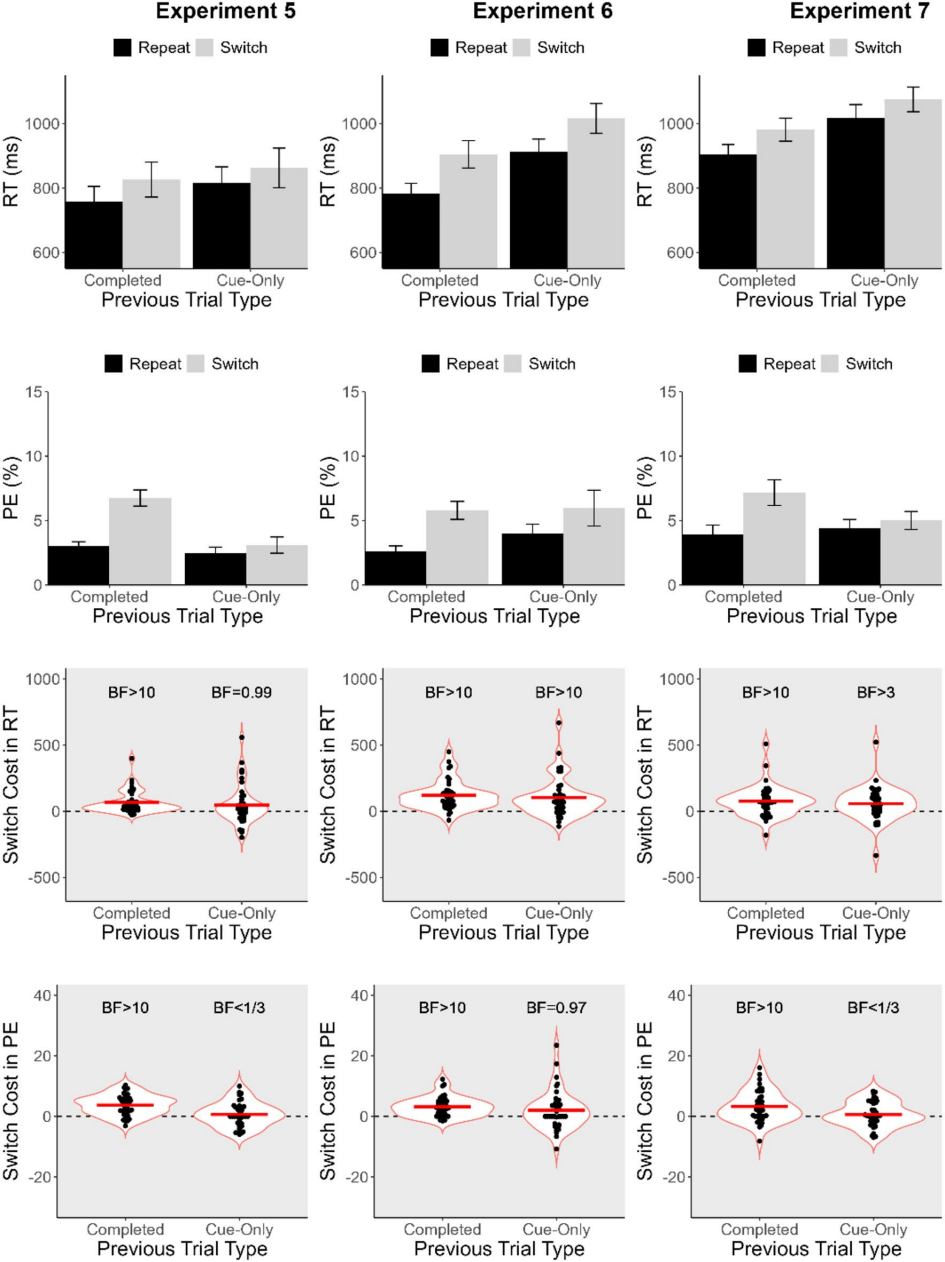
Mean response time (RT), percentage error (PE), switch cost in RT, and switch cost in PE in Experiments 5–7. Error bars in the bar graphs represent one standard error of the means. Horizontal lines within the violin plots represent the grand means, and BFs are the corresponding Bayes factors. BF > 10 or > 3 indicates the presence of a switch cost; BF < 1/10 or < 1/3 indicates evidence for the absence of a switch cost; everything else indicates inconclusive evidence

Mean RT and PE were computed for each participant and were submitted to separate 2 (Previous Trial Type; completed vs. cue-only) x 2 (Task Sequence; task-repeat vs. task-switch) ANOVAs. Task repeat trials were those for which the task cue was the same as on the preceding trial, and task-switch trials were those for which the task cue was different from the preceding one. Regardless of whether the interaction between the two variables was significant, follow-up t-tests were conducted to examine the difference between task repeat and task switch trials (switch cost) after completed and cue-only trials, with Bonferroni-corrected alpha (= 0.025). For each of these switch costs, we computed a Bayes factor (BF) based on a two-tailed one-sample *t*-test against the null hypothesis that a switch cost was absent (= 0). The default prior of the BayesFactor R package (Morey & Rouder, 2022) was used for all calculations of BFs^2^. BF greater than 1 would indicate evidence supporting the presence of a switch cost, whereas BF less than 1 would indicate evidence supporting the absence of a switch cost. We adopted the following criteria to interpret BFs (Jeffreys, 1961). For the presence of a switch cost, BF greater than 3 was taken as moderate evidence and BF greater than 10 as strong evidence; for the absence of a switch cost, BF less than 0.33 was taken as moderate evidence and BF less than 0.10 as strong evidence. BF between 0.33 and 3 was taken as inconclusive or no evidence for either the absence or presence of a switch cost. The ANOVA results are summarised in Table 2, and mean RT, PE, and switch costs, as well as the corresponding BFs, are summarised in Fig. 3 for Experiments 1–4 and in Fig. 4 for Experiments 5–7. Table 1 also summarizes the presence and absence of switch costs after completed and cue-only trials in Experiments 1–7. All analyses and visualization were carried out in R Studio (R Core Team, 2021) with the following packages: tidyverse (Wickam et al., 2019), BayesFactor (Morey & Rouder, 2022), afex (Singmann et al., 2023), multcomp (Hothorn et al., 2008), emmeans (Lenth, 2022), ggbeeswarm (Clarke et al., 2022), and ggpubr (Kassambara, 2022).

**Table 2:** ANOVA results for response time and percentage error in experiments 1–7

Factors	df	Response Time				Percentage Error			
		MSE	F	*p*	η_*p*_^2^	MSE	F	*p*	η_*p*_^2^
		*Experiment 1*							
Previous Trial Type (PTT)	1, 38	**4410.97**	**48.27**	**< 0.001**	**0.559**	13.05	< 1	0.727	0.003
Task Sequence (TS)	1, 38	**1390.82**	**39.56**	**< 0.001**	**0.510**	**11.71**	**25.80**	**< 0.001**	**0.404**
PTT x TS	1, 38	**1479.16**	**60.83**	**< 0.001**	**0.615**	8.73	1.80	0.188	0.045
		*Experiment 2*							
PTT	1, 38	**3711.11**	**52.80**	**< 0.001**	**0.581**	8.73	1.18	0.283	0.030
TS	1, 38	**1523.76**	**20.87**	**< 0.001**	**0.355**	**7.65**	**28.44**	**< 0.001**	**0.428**
PTT x TS	1, 38	**1719.52**	**49.34**	**< 0.001**	**0.565**	5.92	4.02	0.052	0.096
		*Experiment 3*							
PTT	1, 39	**5968.28**	**11.83**	**< 0.001**	**0.233**	11.68	< 1	0.551	0.009
TS	1, 39	**1998.32**	**17.46**	**< 0.001**	**0.309**	**10.74**	**19.25**	**< 0.001**	**0.330**
PTT x TS	1, 39	**1731.68**	**34.21**	**< 0.001**	**0.467**	**4.83**	**38.36**	**< 0.001**	**0.496**
		*Experiment 4*							
PTT	1, 38	3375.30	2.85	0.100	0.070	13.81	< 1	0.564	0.009
TS	1, 38	**2244.75**	**14.07**	**< 0.001**	**0.270**	**15.89**	**19.19**	**< 0.001**	**0.336**
PTT x TS	1, 38	**1641.07**	**25.78**	**< 0.001**	**0.404**	**8.72**	**9.08**	**0.005**	**0.193**
		*Experiment 5*							
Previous Trial Type (PTT)	1, 38	**11776.46**	**7.14**	**0.011**	**0.158**	**7.82**	**21.80**	**< 0.001**	**0.365**
Task Sequence (TS)	1, 38	**9671.31**	**13.45**	**< 0.001**	**0.261**	**9.74**	**18.89**	**< 0.001**	**0.332**
PTT x TS	1, 38	5285.32	< 1	0.383	0.020	**3.83**	**24.73**	**< 0.001**	**0.394**
		*Experiment 6*							
PTT	1, 39	**13339.64**	**43.83**	**< 0.001**	**0.529**	22.77	1.02	0.319	0.025
TS	1, 39	**11805.16**	**42.65**	**< 0.001**	**0.522**	**17.99**	**14.89**	**< 0.001**	**0.276**
PTT x TS	1, 39	5709.54	< 1	0.448	0.015	8.20	1.63	0.209	0.040
		*Experiment 7*							
PTT	1, 38	**9280.90**	**45.08**	**< 0.001**	**0.543**	10.57	2.53	0.120	0.062
TS	1, 38	**6089.45**	**28.49**	**< 0.001**	**0.428**	**10.74**	**13.27**	**< 0.001**	**0.259**
PTT x TS	1, 38	9111.24	< 1	0.536	0.010	**10.84**	**6.36**	**0.016**	**0.143**

Note: Bold indicates a significant effect with alpha = 0.05

### Experiment 1

The present experiment equated the designs of cue-only and completed trials as closely as possible. To do so, we first designed a typical cued task-switching procedure and, then, removed a target on half of the trials, which served as cue-only trials. The other half served as completed trials. If preparation is responsible for producing the switch cost that has been seen many times before (following completed trials), the costs obtained after cue-only and completed trials should be similar. The percentage of discarded trials was 4.71% in this experiment.

For RT, there were significant main effects of Previous Trial Type and of Task Sequence. Responses were generally faster after completed trials (*M* = 686 ms) than after cue-only trials (*M* = 760 ms), and on task-repeat trials (*M* = 705 ms) than on task-switch trials (*M* = 742 ms). There was also an interaction between these two variables, indicating significant differences between switch costs after completed and cue-only trials. There was a significant switch cost after completed trials (*M* = 86 ms, *p* <.001), but there was no significant switch cost after cue-only trials (*M* = − 11 ms, *p* =.140). BF provided strong evidence for a switch cost after completed trials, but no evidence after cue-only trials. For PE, there was a significant main effect of Task Sequence, but that of Previous Trial Type was not significant. Responses were more accurate on task-repeat trials (*M* = 2.65%) than on task-switch trials (*M* = 5.44%). Unlike RT, the interaction between the two variables was not significant; switch cost was 3.42% after completed trials (*p* <.001) and 2.15% after cue-only trials (*p* =.004). After both completed and cue-only trials, BFs provided strong evidence for a task-switch cost.

While a significant switch cost was obtained after completed trials both in RT and PE, it was not significant after cue-only trials in RT but was so in PE. The presence of a switch cost in PE after cue-only trials indicates that there was still an influence of task switching on performance, but the lack of a switch cost in RT still contrasts strikingly with the presence of a substantial RT switch cost following completed trials. The absence of a switch cost in RT following cue-only trials would be a surprising result if the switch costs following cue-only and completed trials both originated from the same process, that is, the processes during the preparation interval including the presentation of a task cue followed by a blank display up until the target’s appearance on a completed trial, irrespective of what happens after that (e.g. target onset on completed trials or the additional blank period on cue-only trials). These results, therefore, suggest that whether or not a subsequent switch cost occurs depends on more than simply the occurrence of that interval during which task preparation could take place, but on other factors. The subsequent experiments aimed to investigate which factors might be critical for finding a significant switch cost after cue-only trials.

### Experiment 2

Experiment 2 focused on the proportions of completed and cue-only trials. In previous studies (Lenartowicz et al., 2011; Swainson et al., 2017, 2021), cue-only trials only occupied a minority of trials (17–30%), whereas cue-only and completed trials occurred with an equal probability in Experiment 1. A small proportion of cue-only trials was assumed to be important because participants might be discouraged from preparing for the incoming task if there is a high likelihood that a target will never have to be responded to (cf. Kleinsorge & Gajewski, 2004). In Experiment 2, cue-only trials only occurred on 25% of trials, which was the same proportion of cue-only trials used by Lenartowicz et al. The percentage of discarded trials was 4.92% in Experiment 2.

For RT, main effects of Previous Trial Type and of Task Sequence were significant. Responses were faster after completed trials (*M* = 640 ms) than after cue-only trials (*M* = 711 ms), and on task-repeat trials (*M* = 661 ms) than on task-switch trials (*M* = 690 ms). As in Experiment 1, the interaction between the two variables was also significant. The switch cost was significant after completed trials (*M* = 75 ms, *p* <.001), but not after cue-only trials (*M* = − 18 ms, *p* =.22). BF provided strong evidence for a switch cost after completed trials but no evidence for a switch cost after cue-only trials. For PE, there was a main effect of Task Sequence, but that of Previous Trial Type was not significant. Responses were more accurate on task-repeat trials (*M* = 3.07%) than on task-switch trials (*M* = 5.43%). The interaction between the two variables was just above the significance level; the switch cost was 3.14% after completed trials (*p* <.001) and was 1.58% after cue-only trials (*p* =.026), which again just missed the Bonferroni-corrected alpha (= 0.025). Again, as in RT, BF provided strong evidence for a switch cost after completed trials but no evidence for a switch cost after cue-only trials. Therefore, the proportion of completed and cue-only trials alone does not account for the lack of a switch cost after cue-only trials in RT in Experiment 1.

### Experiment 3

In the cued task-switching literature, it has been suggested that a short task-cue duration would encourage participants to prepare for the incoming task more (e.g., Verbruggen et al., 2007; Yamaguchi & Proctor, 2011). It is possible that the long task-cue duration in Experiments 1 and 2 discouraged participants from preparing for the incoming task until the target appeared, which would explain why the switch cost was absent only after cue-only trials but not after completed trials. Therefore, the present experiment reduced the duration of task cue presentation from 1000 ms to 200 ms. To maintain the preparation interval (which includes the durations of task cue and the following blank display), the duration of the following blank display was increased from 500 ms to 1300 ms, so that the preparation interval was 1500 ms as in Experiments 1 and 2. In Experiment 3, the percentage of discarded trials was 5.74%.

For RT, main effects of Previous Trial Type and of Task Sequence were significant. Responses were faster after completed trials (*M* = 732 ms) than after cue-only trials (*M* = 774 ms), and on task-repeat trials (*M* = 739 ms) than on task-switch trials (*M* = 768 ms). The interaction between the two variables was also significant; the switch cost was significant after completed trials (*M* = 68 ms, *p* <.001), but not after cue-only trials (*M* = − 9 ms, *p* =.390). BF provided strong evidence for a switch cost after completed trials, but it now provided moderate evidence *against* a switch cost (i.e., evidence for the absence of a switch cost) after cue-only trials. For PE, there was a significant main effect of Task Sequence, but not of Previous Trial Type. Responses were more accurate on task-repeat trials (*M* = 3.54%) than on task-switch trials (*M* = 5.81%). Unlike Experiments 1 and 2, the interaction between the two variables was significant. The switch cost was significant after completed trials (*M* = 4.43%, *p* <.001), but not after cue-only trials (*M* = 0.12%, *p* =.860). As in RT, BF provided strong evidence for a switch cost after completed trials but evidence against a switch cost after cue-only trials. Therefore, the long task cue duration was not responsible for the absence of a switch cost in RT after cue-only trials in Experiments 1 and 2.

### Experiment 4

In all of the previous studies using cue-only trials, the cue-only trials terminated after a blank display that followed a task cue– i.e., they were identical to the pre-target part of a completed trial (Lenartowicz et al., 2010; Swainson et al., 2017; Swainson et al., 2021). In contrast, cue-only trials in Experiments 1–3 of the present study included an additional blank interval after the task cue in place of the target presentation part of completed trials. This additional blank interval might have allowed the conditions for a potential switch cost to dissipate before the next trial started. Therefore, the present experiment removed this additional interval. The percentage of discarded trials was 6.53% in Experiment 4.

For RT, a main effect of Task Sequence was significant, but that of Previous Trial Type was not. Responses were faster on task-repeat trials (*M* = 724 ms) than on task-switch trials (*M* = 753 ms). Unlike Experiments 1–3, responses were no longer significantly faster after completed trials (*M* = 731 ms) than after cue-only trials (*M* = 746 ms), which may be due to the removal of the additional blank after the preparation interval on cue-only trials (but see Experiments 5–7 for which the blank display was removed but RT was still faster after completed trials than after cue-only trials). The interaction between the two variables was still significant; the switch cost was 61 ms (*p* <.001) after completed trials and − 4 ms (*p* =.700) after cue-only trials. BF provided strong evidence for a switch cost after completed trials and moderate evidence against a switch cost after cue-only trials. For PE, there was also a main effect of Task Sequence, but not of Previous Trial Type. Responses were more accurate on task-repeat trials (*M* = 4.20%) than on task-switch trials (*M* = 7.00%). The interaction between the two variables was also significant. As in Experiment 3, the switch cost was significant after completed trials (*M* = 4.22%, *p* <.001) but not after cue-only trials (*M* = 1.37%, *p* =.120). BF provided strong evidence for a switch cost after completed trials, but it provided no evidence for a switch cost after cue-only trials. These results suggest that the long blank display after the task cue on cue-only trials was not solely responsible for the absence of a switch cost in the previous experiments.

### Experiment 5

One of the factors in the four experiments that also differed from previous studies was that there was a response deadline on completed trials. Lenartowicz et al. (2011) reported having no response deadline on completed trials, and other studies (Swainson et al., 2017, 2021) followed this practice. Therefore, Experiment 5 prolonged the response deadline used in the preceding experiments to 10 s, which we believed would impose little time pressure upon participants and allow very slow responses if necessary. The percentage of discarded trials was 4.73%.

For RT, main effects of Previous Trial Type and of Task Sequence were significant. Responses were faster after completed trials (*M* = 792 ms) than after cue-only trials (*M* = 839 ms), and on task-repeat trials (*M* = 787 ms) than on task-switch trials (*M* = 845 ms). Unlike the preceding experiments, however, the interaction between the two variables was not significant, suggesting little evidence for a difference in the switch cost after completed trials compared with that after cue-only trials. Nevertheless, while the switch cost was significant after completed trials (*M* = 68 ms, *p* <.001), it was still non-significant after cue-only trials (*M* = 48 ms, *p* =.056). BF also provided strong evidence for a switch cost after completed trials and no evidence after cue-only trials. For PE, main effects of Previous Trial Type and of Task Sequence were significant. Responses were more accurate after cue-only trials (*M* = 2.79%) than after completed trials (*M* = 4.88%), which had not been observed in the preceding experiments. Reponses were still more accurate on task-repeat trials (*M* = 2.75%) than on task-switch trials (*M* = 4.92%). The interaction between the two variables was also significant; the switch cost was significant after completed trials (*M* = 3.73%, *p* <.001) but not after cue-only trials (*M* = 0.61%, *p* =.340). BF provided strong evidence for a switch cost after completed trials but moderate evidence against a switch cost after cue-only trials.

Unlike the preceding experiments, the interaction between Task Sequence and Previous Trial Type was no longer significant in RT in the present experiment. However, the switch cost was still absent after cue-only trials, suggesting that the use of a response deadline was not responsible for the lack of a significant switch cost after cue-only trials in the preceding experiments.

### Experiment 6

We have so far kept the 1500-ms preparation interval for all experiments, which is on the longer side of the scale used in the previous studies. For example, Lenartowicz et al. used preparation intervals of 350 ms and 1250 ms and found that the switch cost following cue-only trials was reduced from 75 ms when the preceding and current trials had the 350-ms preparation interval to 25 ms when both trials had the 1250-ms preparation intervals, whereas the switch cost after completed trials was only reduced from 78 ms to 53 ms in these cases (although these specific differences were not compared statistically). This pattern suggests that the switch cost after cue-only trials might be overcome more quickly than that after completed trials (see also Swainson et al., 2021). Thus, we shortened the interval from 1500 ms to 300 ms (i.e., 200-ms task-cue duration and 100-ms blank display) in the present experiment. Note that the present experiment reduced the preparation interval uniformly for all trials, not only for completed trials following a cue-only trial. In previous studies, the use of a long preparation interval for cue-only trials was assumed to be important to allow sufficient preparation to take place on these trials. If this is the case, the switch cost could still be absent after cue-only trials in the present study because all trials had a short preparation interval. The percentage of discarded trials was 4.69% in this experiment.

For RT, there were main effects of Previous Trial Type and of Task Sequence. Responses were faster after completed trials (*M* = 844 ms) than after cue-only trials (*M* = 965 ms), and on task-repeat trials (*M* = 848 ms) than on task-switch trials (*M* = 905 ms). The interaction between the two variables was not significant; the switch cost was 121 ms after completed trials (*p* <.001) and 103 ms after cue-only trials (*p* <.001). BF also provided strong evidence for a switch cost after completed and cue-only trials. For PE, a main effect of Task Sequence was significant, but not that of Previous Trial Type. Responses were more accurate on task-repeat trials (*M* = 3.29%) than on task-switch trials (*M* = 5.88%). The interaction between the two variable was not significant; the switch cost was 3.17% after completed trials (*p* <.001) and 2.01% after cue-only trials (*p* =.057). BF provided strong evidence for a switch cost after completed trials but no evidence after cue-only trials.

For the first time in the series, the present experiment yielded large switch costs in RT both after completed trials and after cue-only trials. Therefore, a short preparation interval appears to be a critical factor to yield a switch cost (in RT) after cue-only trials. Interestingly, PE did not produce a switch cost after cue-only trials, which is an opposite pattern to those we observed in Experiment 1 (for which a switch cost was obtained after cue-only trials in PE but not in RT).

### Experiment 7

In this final experiment, we tested whether a switch cost could be obtained after cue-only trials when cue-only and completed trials were equally likely to occur as in Experiment 1. We kept the short preparation interval from Experiment 6. We felt this experiment was necessary because all previous studies assumed that a smaller proportion of cue-only trials was important to detect switch cost after cue-only trials. The percentage of discarded trials was 4.90% in this experiment.

For RT, there were main effects of Previous Trial Type and of Task Sequence. Responses were faster after completed trials (*M* = 943 ms) than after cue-only trials (*M* = 1047 ms), and on task-repeat trials (*M* = 962 ms) than on task-switch trials (*M* = 1028 ms). The interaction between these variables were not significant. The switch cost was 76 ms after completed trials (*p* <.001) and 57 ms after cue-only trials (*p* <.001). BF provided strong evidence for a switch cost after completed trials and moderate evidence for a switch cost after cue-only trials. For PE, there was a main effect of Task Sequence, but not of Previous Trial Type. Responses were more accurate after task-repeat trials (*M* = 4.18%) than after task-switch trials (*M* = 6.09%). The interaction between the two variables was significant; the switch cost was significant after completed trials (*M* = 3.24%, *p* <.001) but not after cue-only trials (*M* = 0.58%, *p* =.380). BF provided strong evidence for a switch cost after completed trials and moderate evidence *against* a switch cost after cue-only trials.

The present experiment also yielded a switch cost after cue-only trials in RT both after completed trials and after cue-only trials. The switch cost in PE was absent after cue-only trials, which also agreed with the outcome of Experiment 6. The present results showed that a switch cost can be obtained after cue-only trials when cue-only trials occurred as frequently as completed trials, which provides an important implication for experimental design using cue-only trials.

## General discussion

The finding that cue-only trials could produce a significant switch cost in a cued task-switching procedure has offered an important theoretical implication as to the source of the switch cost (Lenartowicz et al., 2011). In contrast to the go/nogo procedure used by Schuch and Koch (2003), cue-only trials remove the need to present an extra stimulus (i.e., a nogo signal) that could have interfered with cognitive processes that were responsible for a switch cost (Lenartowicz et al., 2011; Yamaguchi & Swainson, 2024). Hence, the presence of a switch cost after cue-only trials provided clear evidence that task preparation is sufficient to generate a switch cost. However, there have only been a handful of studies using cue-only trials, and much is yet to be known about the nature of a switch cost after cue-only trials. The present study carried out a series of seven experiments to examine several untested assumptions made in the previous studies using cue-only trials and aimed to pinpoint which task parameters are critical to obtaining a switch cost after cue-only trials in comparison to those required to obtain a switch cost after completed trials. A striking result from these seven experiments is that the switch cost was always significant in RT and PE after completed trials, but the switch cost after cue-only trials was obtained only in some of the experiments, and it was never obtained in both RT and PE at the same time. Therefore, there are clear differences between completed and cue-only trials as to when the switch cost follows them.

Experiment 1 matched the conditions of cue-only and completed trials as closely as possible by simply removing the target on cue-only trials such that the two types of trial maintained similar total duration and an equal frequency; cue-only trials were also allowed to repeat in the experiment. The results failed to show a significant switch cost after cue-only trials in RT, although the switch cost was significant after cue-only trials in PE. The results indicated that the influence of cue-only trials differed from that of completed trials for which the switch costs in RT and PE were both significant after completed trials. The subsequent experiments followed up these outcomes to find the conditions required to obtain a switch cost (especially in RT) following cue-only trials.

Previous studies have included less frequent cue-only trials than completed trials, with the assumption that frequent cue-only trials might discourage participants from engaging with advance preparation. Hence, Experiment 2 made cue-only trials less frequent than completed trials. Nevertheless, the results showed no switch cost after cue-only trials in RT or in PE, whereas both RT and PE yielded significant switch costs after completed trials. A short task-cue presentation time has also been shown to encourage advance preparation, so Experiment 3 shortened the presentation time of a task cue. Again, no switch cost was obtained after cue-only trials in RT or in PE; after completed trials, both RT and PE yielded switch costs. The results of Experiments 2 and 3 seem to suggest that the discrepancy between cue-only and completed trials was not due to a lack of incentive to prepare the cued task early within the preparation interval. Experiment 4 further removed the extra blank display after a task cue on cue-only trials (that had been used to match the trial duration with completed trials), which might have caused the effects of processing that took place on the cue-only trial to dissipate before any resulting switch cost could be measured on the subsequent trial. There was again no significant switch cost after cue-only trials in RT or in PE.

The use of a response deadline is a relatively common practice in cognitive experiments, but most previous studies using cue-only trials did not include it in their procedures (Lenartowicz et al., 2011; Swainson et al., 2017; Swainson et al., 2021; but see Brass & van Cramon, 2004). One reason for using a response deadline here was to ensure a fixed maximum duration of completed trials, enabling us to match this aspect between cue-only and completed trials in Experiment 1. Although this design made the duration of cue-only trials longer on average than the completed trials, Experiment 4 demonstrated that this was not the reason for the absence of the switch cost after cue-only trials because cue-only trials were terminated after the preparation interval in that experiment. However, it might have been that the use of a response deadline had prevented the switch cost in RT from emerging after cue-only trials in Experiments 1–4. In order to examine whether that was the case, Experiment 5 prolonged the response deadline to 10 s, which is far longer than typical RTs obtained in the procedure and thus should not impose any time pressure or restrictions in task performance. The results suggested that the lengthening of the response deadline might have influenced the switch cost after cue-only trials, but not enough for the cost to reach statistical significance.

Previous studies have shown that switch costs after cue-only trials are larger when the preparation interval is short than when it is long (Lenartowicz et al., 2011; Swainson et al., 2017, 2021). Experiment 6 shortened the preparation interval to 300 ms (200 ms task-cue presentation and 100 ms blank) as opposed to 1500 ms. The results showed a significant switch cost after cue-only trials in RT for the first time in the present series of experiments; the switch cost after cue-only trials was not significant in PE. It should be highlighted that the preparation interval was shortened not only for completed trials that followed a cue-only trial but also for the cue-only trials themselves. This is important because some previous studies assumed that a long preparation interval for cue-only trials would be necessary for a switch cost to be obtained on the following completed trial. Finally, Experiment 7 used an equal proportion of cue-only and completed trials, again, to examine the influence of trial proportion on the presence of switch costs following each type of trial. The results replicated the significant switch cost after cue-only trials in RT and the non-significant cost in PE that was seen in Experiment 6. Therefore, the results of Experiments 6 and 7 challenged two assumptions made in previous studies using cue-only trials: that the preparation interval on cue-only trials should be long enough that advance task preparation can take place and that the proportion of cue-only trials should be low enough not to discourage advance preparation from taking place. It may be that a switch cost after cue-only trials does not depend on advance task preparation (e.g., because of faster cue encoding when cues repeat; Logan & Bundesen, 2003) or that advance task preparation occurs regardless of these task parameters. It may be that a task cue automatically activates a relevant task-set, so that it does not depend on trial proportion or a long interval on a cue-only trial. It should be noted that the switch cost was not significant in PE in these two experiments.

We focused mainly on the switch costs in RT as the driving force of the current study because RT is usually a more sensitive measure and has been the main dependent variable to examine cognitive control processes in the task switching literature (e.g., Brass & von Cramon, 2004; Kleinsorge & Gajewski, 2004; Lenartowicz et al., 2011; Logan & Bundesen, 2003; Yamaguchi & Proctor, 2011). Theoretical reviews of the task-switching literature are based primarily on RT data and seldom (if ever) mention any data from response error (e.g., Kiesel et al., 2010; Monsell, 2003; Vandierendonck et al., 2010). In fact, the present study showed that the switch cost following cue-only trials was significant in PE only in Experiment 1, but not in the subsequent six experiments. Therefore, we conclude that the present series of experiments indicate collectively that the use of a short preparation interval is important for determining the presence of a switch cost after cue-only trials, and that researchers who wish to design paradigms that generate a switch cost following cue-only trials need not require either that the cue-only trials are long in duration or that they occur at a low frequency. Furthermore, whereas the previous studies did not allow cue-only trials to occur consecutively, the present series of experiments allowed it, indicating that such a design feature was not required either.

### How is task switching after cue-only trials different from task switching after completed trials?

Our recent work asked how switch costs after cue-only and completed trials differed (Swainson et al., 2024), by using a double-registration paradigm of Arrington et al.’s (2007) in which participants first responded to the task cue (cue-response) and then to the target (target-response) on each trial. We found a remarkable similarity between the nature of the task-switch costs following cue-only trials and completed trials, in that task-switching affected target-responses rather than cue-responses in both cases. Nevertheless, the study also showed that the task-switch cost was significantly larger following completed trials than it was following cue-only trials. The current study shows a more striking difference between the switch costs following cue-only versus completed trials: when measured in RT, the switch cost following completed trials was present in all of the experiments regardless of preparation interval, whereas a switch cost in RT following cue-only trials was obtained only in Experiments 6 and 7 in which preparation intervals were short. It is often seen that part of the switch cost persists even with a long preparation interval. This *residual switch cost* is interpreted as indicating that there is an intrinsic limit to advance preparation in overcoming the cost of task switching (e.g., Meiran & Chorev, 2005; Nieuwenhuis & Monsell, 2002). Our observations imply that there was no residual switch cost after cue-only trials in these experiments, suggesting the possibility that the switch cost after cue-only trials may be qualitatively different from that after completed trials. Rogers and Monsell (1995) attributed the presence of the residual switch cost to the need for an exogenous reconfiguration process following target stimulus onset; it may be that there is no need for exogenous reconfiguration following cue-only trials which, by their nature, involve no target. Or it might be that the increased persistence of the switch cost after a completed trial reflects persisting inhibition of the competing task as a consequence of selecting a response to the target on these trials (Schuch & Koch, 2003). A further possibility is that because targets and responses occur on completed trials, they are bound together (Schmidt et al., 2020; Schmidt & Liefooghe, 2016); this binding may contribute to the switch cost measured on the next trial.

Why was the preparation interval needed to be short for a significant switch cost to emerge after cue-only trials in these experiments? Note that this finding was presumably driven by the short preparation interval on a completed trial following a cue-only trial rather than on the preceding cue-only trial itself, because previous studies still found significant switch costs following cue-only trials when the cue-only trials had a long preparation interval (e.g., Lenartowicz et al., 2011; Swainson et al., 2017, 2021). A likely reason is that a short preparation interval on the current trial provides little time during which the old task could decay or be overcome before a new target occurs, which would result in a larger switch cost. It is interesting to observe that in Experiments 6 and 7, in which the preparation interval was short, the switch cost after cue-only trials was similar in magnitude to, but not smaller than, the switch cost after completed trials. If responding to the target had enhanced the switch cost, we would have obtained a larger switch cost after completed trials than after cue-only trials, as in the aforementioned study (Swainson et al., 2024). Thus, if we assume that the switch cost results from the effect of a task-set having been activated on the preceding trial, then the present results seem to suggest that responding to the target can prolong the activation of a task-set to make its influence more persistent over a longer period but that it may not necessarily increase the activation to enlarge the influence. For instance, if task-set activation is a sigmoid function that reaches an asymptote (see Cohen et al., 1990; Logan et al., 2015), there would be a ceiling on the magnitude of a switch cost that sets a limit to how much a task-set could be activated.

Alternatively, it could be that performing a task can increase the subsequent switch cost, but that other factors counteracted that effect in this study and prevented the increase from being seen. For instance, as argued by Swainson et al. (2021), participants could be less ready to switch following cue-only trials than they are following completed trials. Overall, task switching is required less frequently after a task cue than after a target (because at least 50% of task cues are followed by targets rather than by another cue, and only task cues can signal a task switch), potentially making participants less prepared for task switching after a task cue and, thus, increasing the switch cost after cue-only trials. Also, it might be that participants find it more difficult to disengage from a task that has not been completed than from one that has (e.g., see Bugg & Scullin, 2013). Such effects could offset a large task-switch cost after completed trials. These possibilities require future scrutiny.

Also, recent studies examined the influence of cue-only trials in lexical decision (Berger, Kunde, et al. 2024) and in a backward inhibition design (Berger, Koch, et al., 2024) and found that the influence only emerged on early trials in a session and then disappeared for later trials. These findings raise the possibility that the task-switch cost following cue-only trials in our own experiments might also have emerged on early trials and then disappeared as participants performed more trials. As a supplementary analysis, we also examined this possibility by calculating moving averages of the switch cost over trials in the present study, but little evidence supporting this possibility was found. Because the present study was not designed to carry out this specific analysis, it would be interesting to find out whether a result similar to the influence of cue-only trials in Berger et al.’s studies is also obtained in cued task-switching in future investigations.

### Reconciling response-based and preparation-based accounts

The present results offer a reconciliation of the two competing proposals from Schuch and Koch (2003) and Lenartowicz et al. (2011). Schuch and Koch proposed that a switch cost is caused by the inhibition of an activated (but not currently relevant) task-set that occurs only when response selection is performed. This proposal was based on their finding that both the switch cost and the N-2 repetition cost (the cost of repeating the task from two trials prior to the current trial, which is thought to reflect an inhibition of a previous task-set when a task switches) were absent after nogo trials. Lenartowicz et al. challenged this account of the switch cost by using cue-only trials and concluded that preparation was sufficient to generate the switch cost. The present results agree with Lenartowicz et al.’s conclusion that processes involved in task preparation are sufficient to generate a switch cost, but they also indicate that the switch cost generated solely by task preparation can be rather fragile and short-lived. A more persistent cost is obtained when a response is made to the target, which is consistent with Schuch and Koch’s account as responding requires selecting the response.

Therefore, in light of the present results, we suggest that there are at least two sources of the switch cost. The first source is an early phase of task-set activation, which starts with encoding of a task cue but may not involve a full activation of a task-set. For example, cue-encoding may create a cue representation that persists and interferes with cue-encoding on the following trial (e.g., Altmann & Gray, 2008; Logan & Bundesen, 2003), and this might be easier to overcome than a fully activated task-set would be as the new task-set dominates the process. Alternatively, Schuch and Koch (2003) proposed that two task-sets can be active in parallel prior to response selection. These two task-sets may not interfere with each other directly (i.e., activation of the new task might not compete with activation of the old task as just described) but by being active concurrently they could potentially still create conflict between different responses, slowing response selection (cf. the response-congruity effect often observed in cued task switching; e.g., Rogers & Monsell, 1995; Yamaguchi & Proctor, 2011). These possibilities could explain the short-lived nature of the switch cost after cue-only trials, as compared to the switch cost after completed trials, as observed in the current set of experiments.

However, the persistent switch cost after completed trials is difficult to explain in terms of concurrently active task-sets alone, because a process that was due to task preparation should be happening after both completed and cue-only trials. Hence, a second source of the switch cost might originate from processes that are required in responding to the target. Schuch and Koch (2003) proposed that inhibition of the old task-set can arise at response selection, creating a cost of disinhibiting that old task-set when it is required again on a subsequent (switch) trial. When there is no target presented, no response-selection process takes place, and there may not be inhibition of the old task-set that would result in a persistent switch cost after cue-only trials. In line with this idea, Prosser et al. (2023) found no evidence of an N–2 repetition cost affecting target responses following cue-only trials in a double-registration design. Alternatively, as discussed above, it might be that other processes that only occur on completed trials, such as exogenous reconfiguration or feature-binding, are what result in the persistent subsequent switch cost. The current views of task switching (e.g., Kiesel et al., 2010; Vandierendonck et al., 2010) are consistent with the existence of the two sources of a switch cost as proposed here.

The present demonstration that the preparation interval for cue-only trials does not need to be long may argue against the assumption that the switch cost after cue-only trials can only be driven by a lengthy task preparation, such as may be required if processes of task-set activation/inhibition or reconfiguration are involved. Instead, it might be that processes that occur at an early stage of preparation and that are less time-consuming but still obligatory can cause a switch cost after cue-only trials. Such processes as cue identification (Logan & Bundesen, 2003) or task identification (Altmann & Gray, 2008) may be sufficient to cause the subsequent switch cost. This proposal is consistent with an earlier finding that the size of the switch cost after cue-only trials was not correlated with the amount of reduction in the switch cost during the preparation interval (Swainson et al., 2017). The question of what specific process(es) involved in task preparation produces a switch cost still remains to be answered in future investigations. Such investigations might usefully separate two aspects of the overall switch cost that relate specifically to the need to switch between cues versus between tasks (see e.g. Logan & Bundesen, 2003), which were not separated in the current study. These aspects of the overall switch cost might potentially require different conditions for their generation and might also behave differently in the presence of a long preparation interval. For instance, task-switch costs following cue-only trials have been shown to persist beyond a preparation interval of 800 ms on the current (completed) trial in previous studies (Swainson et al., 2021, Experiment 1; Swainson et al., 2024). In those studies, task-switch costs excluded cue-switch costs and the cue-only trials themselves had a long preparation interval, were infrequent, and could not occur consecutively; in the later study, cues also required their own responses. It remains to be seen whether different experimental conditions generate different aspects of the switch cost that follows cue-only trials.

While advocating for the two sources of the task-switch cost, one must exercise caution because verifying the existence of two separate cognitive processes responsible for a given phenomenon involves more than simple dissociations of experimental factors (e.g., Dunn & Kirsner, 1988; Sternberg, 2003). In the present series of experiments, we found that the preparation interval was critical to find a significant switch cost after cue-only trials, but it has also been shown previously that the preparation interval influenced the size of the switch cost after completed trials (e.g., Verbruggen et al., 2007). It is, therefore, not the case that the preparation interval functionally dissociated the switch cost after the two types of trials. Our proposal rests on the robustness of the switch cost after completed trials in the series of experiments when it was not obtained or only obtained either in RT or in PE after cue-only trials. An experiment designed specifically to test the two-process model against a single-process model is required to confirm the proposal.

## Conclusion

The present study examined possible contributions of various task parameters to the presence of a switch cost after cue-only trials. The results imply that a switch cost after cue-only trials can be short lived and can dissipate or be overcome quickly, whereas a switch cost after completed trials is more persistent and can be obtained consistently even in conditions for which cue-only trials do not produce a clear switch cost. We propose that there are at least two separate sources of a switch cost: one that is due to processes involved in task preparation, such as priming of cue-encoding, interference between two concurrently active task-sets or a response conflict caused by the concurrent task-sets, which can arise when a task cue is presented; and another that is due to processes involved in responding to the target, such as inhibition of a previous task-set at response selection, exogenous task-set reconfiguration, or feature binding, which can only arise after a target stimulus is presented. Because cue-only trials only involve the former source but completed trials involve both, the switch cost after cue-only trials can be short-lived and more fragile than that after completed trials. This proposal corroborates contemporary views of the switch cost (Kiesel et al., 2010; Vandierendonck et al., 2010) that also attribute a switch cost to preparatory processes (before a target onset) as well as task performance. The present series of experiments pinned down factors that are, and are not, important for a switch cost after cue-only trials. The proportions of cue-only and completed trials, the length of task-cue presentation, the removal of a long blank after a task cue and the removal of a response deadline were not sufficiently influential to ensure that a significant RT switch cost was present following cue-only trials. In contrast, the length of the preparation interval was found to be a critical factor in the current experiments, such that a significant switch cost was observed in RT when the preparation interval was short. These demonstrations provide a useful recipe to obtain a switch cost after cue-only trials, which potentially helps to reconcile the competing accounts of the origin of a task-switch cost in cued task switching.

## Data Availability

The experimental data and analysis scripts are available for reanalysis purposes from the Open Science Framework project page (https://osf.io/9p2ws/).

## Footnotes

1 We did not exclude trials with long RTs because one of the experimental settings was to use a very long response deadline (Experiments 5–7), following previous studies on cue-only trials (Lenartowicz et al., 2011; Swainson et al., 2017, 2021). Recent studies examining the influence of outliers in RT (Berger & Kiefer, 2021, 2023; Miller, 2023) suggested that different outlier exclusion methods lead to different biases in the results. We also analyzed the data after excluding trials for which RT was greater than 2000 ms, consistent with the response deadline used in Experiments 1–4, which resulted in shorter RTs and smaller switch costs overall but did not alter the main findings.

2 Note that BF is sensitive to the scale parameter (*r*) of a Cauchy distribution used in the BayesFactor package, such that decreasing the scale parameter increases the bias favoring the alternative hypothesis when the effect size is non-zero while increasing the scale parameter increases the bias favoring the null hypothesis when the effect size is zero; the relative biases favouring the null and alternative hypotheses depend on the sample size and the effect size (Hoijtink, 2022). We chose to use the default parameter (*r* =.707) to avoid an ad-hoc decision because the optimal parameter could be decided only after the effect size is known. However, we note that, according to Hoijtink’s analysis, the default parameter would bias against the alternative hypothesis more when the effect size is small whereas it would bias against the null hypothesis more when the effect size is large.
